# A qualitative exploration of the experience of individual-level stigma among adolescents with a chronic illness

**DOI:** 10.1371/journal.pone.0317633

**Published:** 2025-12-18

**Authors:** Joan A. Thomas, Garth Lipps

**Affiliations:** 1 Caribbean Child Development Centre, Consortium for Social Development and Research, The University of the West Indies – Mona Campus, Kingston, Jamaica; 2 Department of Sociology, Psychology and Social Work, The University of the West Indies – Mona, Kingston, Jamaica; University of Haifa Faculty of Social Welfare and Health Sciences, ISRAEL

## Abstract

**Background:**

Much of the research measuring stigma experience related to illness in children and adolescents has been conducted using measures developed from adult perspectives. However, children and adolescents are different from adults developmentally, and stigma is a subjective concept. As the first step in a multi-phase process to develop generic measures of individual-level stigma for use with adolescents with an illness, a qualitative study was conducted to explore the lived experiences of the different forms of individual-level stigma among adolescents with various illnesses.

**Methods:**

Semi-structured interviews, utilizing multiperspectival interpretive phenomenological analysis, were conducted to identify commonalities in the experiences of perceived, enacted, and internalized stigma among adolescents with different chronic conditions recruited from illness-specific clinics at 2 general hospitals and a public clinic.

**Results:**

The adolescents’ experience of perceived stigma was centered on negative treatments they anticipated receiving due to their illnesses, rather than a focus that also included awareness of illness-specific stereotypes, as suggested by the Modified Labelling Theory. Their awareness of illness-specific stereotypes was limited and mainly internalized, manifesting itself as negative self-beliefs. Their fear of negative treatments, also internalized, led them to conceal their illnesses and have negative feelings about themselves. They experienced enacted stigma mainly in interpersonal relationships in overt and subtle ways. Their interpretations of some of the treatments they received from others influenced their experience of enacted stigma. Furthermore, the adolescents’ experience of individual-level stigma was influenced by their level of development, cognitive maturity, and changes in their peer groups. Also, their experience of stigma bore some similarities with adults.

**Conclusions:**

The study found that the adolescents’ experience of the different forms of individual-level stigma bore some similarities with adults as well as there were differences. The differences were due to the influence of social and cognitive development. The findings should inform quantitative assessments of individual-level stigma in adolescents with an illness.

## Introduction

Having to cope with a chronic illness is a common occurrence in the lives of many children and adults around the world [1–3]. Improvements in diagnosis and treatment have shifted the course of many illnesses from life-threatening to controllable and long-lasting, requiring that these illnesses be managed continuously over an extended period [1,2].

Owing to the longevity achievable due to improvement in treatment for many chronic illnesses, stigma related to illness has risen in importance as it contributes to the burden of having to cope with the illnesses and thus influences psychosocial outcomes [4–6]. Stigma has received increasing attention in the literature as an important determinant of several negative psychosocial outcomes in people with an illness. This work, however, has primarily been conducted for adults with chronic illnesses.

Many of the chronic illnesses that have been associated with stigma in adults, such as sickle cell, diabetes, asthma, epilepsy, and mental illness, have their onset in childhood or adolescence [7,8]. Past attempts at measuring children and adolescents’ stigma experiences have largely been based on measures developed for adults or from the perspectives of proxies [9–15]. The literature suggests that conceptualizing illness-related stigma based on adults’ perspectives cannot sufficiently describe the experiences of stigma for children and adolescents. This is primarily because children’s and adolescents’ social and emotional contexts are different from adults due to differences in their levels of cognitive and social development [16–22]. Furthermore, the experience of stigma is inherently subjective and socially constructed [6]. Therefore, for accurate conceptualization and assessment, the lived experiences of children and adolescents must be central to the study of stigma among them.. This necessity requires the utilization of a qualitative approach, which allows for an in-depth exploration of personal meaning and experience [16,19,21]. The aim of this research is to describe commonalities in the experience of individual-level stigma from the lived experiences of adolescents with a variety of illnesses. Addressing this question is important because the findings will fill gaps in knowledge on how stigma develops from childhood to adulthood. Additionally, the findings will help to better understand and measure the development of stigma across the life span, which is essential for informing the creation of developmentally tailored anti-stigma interventions..

### The conceptualization of stigma

The conceptualization of stigma is rooted in the work of Erving Goffman [23], who defined it as an attribute or a characteristic that differentiates an individual from others and causes them to be seen as less desirable*—*a “tainted” or “discounted” person*—*in the minds of others. Subsequent definitions of stigma have adapted or elaborated Goffman’s definition in an effort to describe the nature, sources, and consequences of stigma, often moving to a more structural view [24,25]. Most notably, Link & Phelan [24] expanded Goffman’s conceptualization by taking into consideration the influence of social processes and cultural context on an individual’s stigma experience. They framed stigma as a process where an undesirable attribute or characteristic becomes associated with a negative identity through labeling, stereotyping, separation, and discrimination, all of which are enforced within a context of power to create unequal outcomes.

A chronic illness serves as the undesirable attribute that triggers the entire social process of stigma: the illness label is linked to negative stereotypes as it is seen as a deviation from what society perceives as being healthy [26,27]. This results in prejudice, discrimination, and status loss, which collectively lead to social exclusion and other poor outcomes for individuals with the illness.

### Stigma in children and adolescents with a chronic illness

Research has associated stigma with a wide variety of chronic illnesses, including weight issues [28], diabetes [29], HIV [30], epilepsy [31], asthma [32], sickle cell disease [33], cystic fibrosis [34], and mental illness [35,36]. Most of this research on stigma, however, has been done with adults [14]. Many of these chronic illnesses also occur in children and adolescents. In fact, several of them have their origin during childhood, for example, asthma, sickle cell disease, epilepsy, and cystic fibrosis [7,8]. Several others have an increasing incidence during adolescence, for example, mental illness, HIV, weight issues, and diabetes [7,8]. However, the stigma experienced by children and adolescents with many illnesses has received less attention in research compared with adults with the conditions. Less is known about how stigma manifests itself in the lives of many of these children and adolescents, and the consequences of this for them. A gap in information regarding the stigma experiences of children and adolescents with several specific health conditions has been noted by the researchers carrying out work concerning the specific conditions, for example, mental illness [37,38], HIV [18], and chronic pain [39,40].

### Why care about the stigma experienced by children and adolescents with a chronic illness?

#### Children’s and adolescents’ awareness of stigma.

The stigma experienced by children and adolescents with a chronic illness is important to consider because this developmental period is when individuals become aware of and internalize stigmatizing ideas associated with an illness or other socially “deviant” characteristics [23,24,36,41]. Stigmatizing views regarding an undesirable attribute, such as a health condition, are learned early in life through socialization and are widely understood within a culture, including by those who possess the attribute. These views are typically acquired from multiple sources, including family lore, peer relations, personal experience, and media portrayals [36,42,43]. Research indicates that children acquire the cognitive capability to identify differences by five years old [19,44,45]. By ten years old, most children are aware of cultural stereotypes related to stigmatized characteristics, and children who are subjects of stigmatization may acquire this awareness at an even younger age [41].

#### Development of children and adolescents, and the experience of stigma.

The experience of stigma is also salient to consider for children and adolescents because they must cope with the demands of normal developmental challenges simultaneously with the inherent difficulties of managing their health condition [46]. This dual burden suggests that their experience of stigma is likely to differ from that of adults with similar conditions. Their unique experience is further complicated by the fact that there are stages to their development that will likely significantly influence both the nature and the severity of the stigma encountered [22,47]. Unlike adults, whose developmental tasks are largely finalized, for children and adolescents, this is characterized by a period of rapid and continuous physical, cognitive, and social change [3,17,18,22].

The influence of stigma will be mainly felt across two of these developmental domains, that is, the cognitive and the social. Cognitive development entails a progression from concrete, sensory understanding in childhood to more abstract and organized reasoning in adolescence [17,48]. The level of cognitive development thus will determine the capacity of children and adolescents to interpret and express their experience of illness-related stigma, since abstract thinking abilities increase with age [18,48]. Simultaneously, social development requires youth to shift focus from parents to peers, form a personal identity and sense of self, and manage a growing preoccupation with their self-image [3, 22, 49-50]. As stigma is primarily experienced in social interactions [51], its presence is likely to interfere with and potentially delay the social development of youth with an illness. This interference may, in turn, negatively affect their long-term psychosocial outcomes [16,20].

### The cultural context of stigma for Jamaican children and adolescents

Culture is defined as the shared values, beliefs, practices, and social norms of a national or local group [52] and hence is central to how stigma is experienced by an individual. In the Jamaican society, culture influences the stigma for adolescents with chronic illnesses through three key avenues.

First, gender roles influence how children and adolescents are expected to think, feel, and behave based on their assigned sex, due to socialization. In Jamaican society, this process socializes males toward “macho” strength (rough, strong, dominant) and females toward “ladylike” gentleness (soft, gentle, modest) [53]. For young individuals with chronic illnesses, the inability to fully embody these strong cultural expectations—such as a male’s perceived physical weakness or a female’s need for extensive care—can significantly exacerbate the stigma they experience.

Second, literature suggests that parents are primarily responsible for their children’s illness management when they are young but should become progressively less involved as the child enters adolescence, only maintaining a supervisory role [54–56]. However, Jamaican parents frequently remain highly involved in their adolescents’ lives, often continuing this involvement well into adulthood. This intense engagement manifests as parents driving them to school, being highly involved in education, and actively attempting to protect them from negative experiences, particularly female adolescents [57]. This extended high parental involvement can distinctly shape a young person’s experience of stigma, potentially leading to feelings of being overprotected or different from peers.

Third, the language and verbal expressions used in a culture shape how the world is experienced by members of that culture. Due to developmental differences, the language used by children, adolescents, and adults may vary, thereby producing differences in perceptions and experiences of stigma [58,59]. For children and adolescents with chronic illnesses, the specific language and social discourse within the Jamaican society will influence how they perceive, articulate, and internalize their experiences of stigma.

### Methodological challenges with research assessing stigma in children and adolescents

Relatively little of the research quantifying the experience of stigma among children and adolescents with a chronic health condition has been grounded in their lived experiences or self-reports. Specifically*,* attempts at measuring their stigma experience have largely been based on the use of measures developed for adults that were subsequently modified, or measures that were developed from the perspectives of proxies, such as parents/caregivers or health professionals [9–15,60]. However, it was noted that this practice is likely problematic as the areas covered in adult measures may not totally capture or readily overlap with the social experiences and contexts of children and adolescents [15,16,21]. Additionally, it was highlighted that stigma is a subjective experience [16]. Therefore, adults’ perspectives or experiences might not be appropriate to conceptualize and understand the experience of stigma for children and adolescents with an illness as their lived experiences are different from adults, both in terms of their understanding of their illness and how they believe they are affected by it. This is because of their social, cognitive, and emotional development [16,18,19]. Stigma assessments in general have been criticized for being “uninformed by the lived experience of the people they study” [24 p. 365].

### Theoretical Framework

An expanded Modified Labelling Theory (MLT) was used as the basis for conducting the research and interpreting the findings. The Modified Labelling Theory [42] is commonly used in the sociological and psychological literature for explaining how people with a health condition experience stigma at the individual-level, and the negative consequences of this for them. The theory, which is based on work with adults, proposed a process of experiencing stigma which emphasizes perceived stigma – an individual’s reaction to negative societal stereotypes, attitudes, and behaviours – highlighting the social and psychological consequences of this for those experiencing stigma. According to them, people with a characteristic that differentiates them from others internalize negative societal conceptions and stereotypes about their characteristic through having an awareness of these negative stereotypes and conceptions and expecting devaluation and discrimination of themselves and others with their characteristic. In response to perceived stigma, they engage in various behaviours (e.g., secrecy, limiting social interaction, educating others, etc.) to protect themselves from possible discrimination and devaluation, which results in them experiencing several social and psychological consequences. The discrimination and devaluation they anticipate, also called enacted, experienced, social or public stigma in the literature, may be overt or subtle negative treatments from others [61–63].

Corrigan and colleagues [35,36,64,65] expanded the Modified Labelling Theory (MLT) by proposing self-stigma as a further reaction to the internalization of negative societal conceptions and stereotypes. According to them, people with a characteristic that differentiates them from others not only have an awareness of negative societal stereotypes about their characteristic and expect devaluation and discrimination, but they also agree that the negative stereotypes are true about people with their characteristic and accept these as true of themselves as well. This agreement with the negative stereotypes and their application to oneself results in a reduction in their self-esteem or self-efficacy. Self-stigma has also been called internalized stigma in the literature [43], and may manifest as various negative beliefs, thoughts, feelings, and behaviours [61,63,66–68].

Although the original work of Link and colleagues [24,42], and Corrigan and colleagues [35,36,64,65] focused on mental illness, the framework has been successfully extended to other illnesses, finding support for the three forms of individual-level stigma [61,69,70]. The aim of the current study was to apply the modified labeling theory and its expansion as a theoretical lens to conceptualize commonalities in the experience of individual-level stigma from the lived experiences of adolescents with various illnesses. Completing this aim will serve as the first step in a multi-phase research project to develop generic measures of individual-level stigma for use with adolescents who have an illness.

## Method

### Study design

A qualitative exploratory study was done, utilizing Interpretive Phenomenological Analysis (IPA) to examine adolescents’ lived experiences of the forms of individual-level stigma. IPA is fundamentally grounded in a constructivist (or interpretivist) epistemology, which asserts that reality is subjective and that knowledge is generated through interpreting how participants make sense of it [71–73]. IPA focuses on exploring how individuals make sense of their lived experiences of a particular phenomenon – in this case, the experience of individual-level stigma among adolescents [74,75]. The study departed from the traditional single case study IPA by adopting a multi-case design, collecting data from six samples (adolescents with different health conditions) to conceptualize the commonalities in the experience of stigma across samples (health conditions). This extension of IPA, which treats each illness group as a distinct case study, follows the guidance of Larkin et al. [76] and the example of McInally and Gray-Brunton [77] regarding multiperspectival IPA, enabling the analysis of a complex, systemic experiential phenomenon while upholding IPA’s commitment to the in-depth analysis of each case first (idiography).

### Participants and inclusion criteria

Six separate convenience samples of adolescents 12–19 years of age with a chronic health condition were included in this study (Table 1). The specific health conditions that were sampled included HIV, sickle cell disease (SCD), endocrine condition (diabetes, thyroid issues, hormonal and weight issues), pulmonary condition (asthma), neurological condition (epilepsy), and mental illness (depression, schizophrenia, and conduct disorder). The samples of adolescents were recruited from illness-specific clinics at the University Hospital of the West Indies (UHWI), Kingston Public Hospital (KPH), and the Comprehensive Health Centre in Jamaica (CHC). These health facilities were the largest of the public facilities that provided access to multiple adolescents with diverse illnesses and were the closest in proximity to the first author, who collected the data. The illness-specific clinics were those that granted permission for the study to collect data from their attendees.

**Table 1:** Demographic characteristics of adolescents.

Characteristics			
**Gender**		**n**	**%**
	Male	30	94. 8
	Female	37	55.2
**Attending school**			
	Yes	64	95.5
	No	3	4.5
**School level**			
	Remedial	1	1.5
	All Age/Junior High/ High	62	92.5
	Post-high	4	6.0
**Type of condition**			
	Sickle Cell Disease (SCD)	18	26.9
	Endocrine condition (diabetes, hypothyroidism, hormonal issues/hormone imbalances, and weight issues)	16	23.9
	Mental illness (depression, schizophrenia, and conduct disorder)	14	20.9
	HIV	7	10.4
	Neurological condition (epilepsy)	8	11.9
	Pulmonary condition (asthma)	4	6.0

Note: n = 67 participants

Inclusion criteria for adolescents to participate in the study were that they were aware of their conditions, had been living with their conditions for at least three months, and were carrying on with day-to-day activities (i.e., attending school or working). Adolescents were excluded if they had just learned of their conditions or had been living with their conditions for less than 3 months or had a cognitive challenge.

### Semi-structured interviews

Data were collected from the study participants in semi-structured interviews on their experiences with their health conditions. The interview guide (see S1 File In-depth interview guide) was developed by the first author with the guidance of the literature [50,78–81]. In the interview, each youth was asked questions about the discovery of their health condition, including the symptoms they experienced and the cause of their condition; experiences with receiving treatment and managing the condition; disclosure of the condition to others; their feelings about having the condition as well as the views and reactions of others regarding their condition; their responses to others’ reactions; and the effects of the condition on their lives. Demographic data were collected from each participant prior to asking them about their illness. The demographic data collected included age, gender, name of illness, whether the participant was attending school or not, the school they were attending or attended, and the participant’s current or last grade.

### Data collection

Ethical approval was obtained for the full research (this phase and the next) from The University of the West Indies (UWI), Mona Campus Research Ethics Committee (MCREC) (#ECP 200, 13/14; approval obtained July 8, 2014) and the Southeast Regional Health Authority (SERHA) Ethics Committee, of the Ministry of Health and Wellness in Jamaica (approval obtained February 12, 2015). Additionally, approvals were obtained from the relevant authorities at the hospitals and clinics from where the study participants were recruited.

Prior to the data collection, the interview questions were piloted with five adolescents with different illnesses to test the questions and their interpretations. Minimal changes were made to the questions as a result. The data collected from the pilot interviews were not included in the final data analysis.

For the data collection, potential adolescents and their parents were identified with the help of care staff during their visits to their illness-specific clinics. All potential participants or their parents were informed about the study and asked if they would take part. If they agreed, each adolescent’s awareness of their illness and other inclusion criteria were confirmed. Written parental informed consent and youth informed assent were obtained for participants under 18 years old, and written youth informed consent was obtained for those participants 18 years and older prior to the data collection.

The recruitment of study participants and the semi-structured interviews were conducted between October 1, 2014 and July 31, 2015, by the first author, who had no pre-existing relationship with the participants. The interviews were mostly done with the participants in the clinics (n = 48 interviews), with some interviews done in participants’ homes or at other locations (n = 12 interviews) or via Skype or telephone (n = 7 interviews). A social worker or counsellor was identified in the clinics and was on hand in case any of the participants became upset during the interviews in the clinics and needed help. The length of the interviews ranged from 30 minutes to two hours. All the interviews were audio-recorded.

### Positionality and reflexivity

The first author worked as a social researcher for over 10 years prior to conducting this research. She became interested in the area following her organization’s prior work on a related area: stigma attitudes and children and adolescents with HIV/AIDS. She recognizes that her gender, social class, and age could influence the data collection process. As such, she made a deliberate attempt to ensure that participants were comfortable with her during the interviews by establishing a trusting communication climate and portraying a nonjudgmental persona.

### Data management and analysis

The Interpretive Phenomenological Analysis (IPA) methodology and its associated philosophical assumptions guided the management and analysis of the qualitative data. The research consisted of six separate illness samples, each of which formed a case study for IPA based on the guidance for multiperspectival IPA [76,77]. The first case study, utilizing adolescents who had sickle cell disease, served to establish the initial analytical foundation for the subsequent five illness groups.

The audio recordings of the semi-structured interviews were first transcribed verbatim by a transcription service. The first author then read and re-read the transcripts to ensure accuracy and to achieve initial immersion in the participants’ accounts. Transcribed texts for each illness were then organized by areas on the interview guide using a spreadsheet, as no specialized software was utilized.

The analysis began with sickle cell disease, the first case study, and followed the established six-step process for IPA, focusing first on the idiographic (in-depth) analysis of each participant’s transcript through a double hermeneutic process, progressing from initial annotation (descriptive, linguistic, and conceptual notes) to the development of emergent themes specific to the participant [71,75]. The emergent themes were then grouped to produce a structure of superordinate and sub-themes for the individual participant. This full idiographic analysis was completed sequentially for all participants within the first case study (sickle cell disease group). The themes for all participants in this first case study (illness group) were then synthesized to produce a final set of themes and sub-themes for the sickle cell disease sample. The same detailed set of IPA analysis procedures was then applied to each of the subsequent five case studies (illness groups). As the analysis progressed, the codes, categories, and sub-themes were continuously compared across case studies to ensure trustworthiness, rigor, and consistency in the process and to ensure they remained grounded in the data [20,82]. New insights that emerged during the analysis were added, and existing thematic categories were refined and revised as necessary to best reflect the data. The Expanded Modified Labelling Theory (MLT) was used as a framework to inform interpretation during the later stages of analysis, helping to organize and interpret emergent themes in relation to the established constructs of perceived, enacted, and self-stigma.

To ensure analytical rigor, the first author discussed the emergent codes, categories, and sub-themes with the second author. Discrepancies were resolved through discussion, often requiring a return to the original transcripts and subsequent revision of the coding to ensure consensus and fidelity to the data. The interpretations and descriptions of the adolescents’ experiences were also with a sample of them (one participant from each illness group) to ensure that the evaluations accurately reflected their experiences [83]. If the adolescents pointed to or identified discrepancies with the descriptions or interpretations of their experiences, the information was modified to reflect the perspectives of the adolescents.

## Results

### Stigma experiences

**Fig 1:**
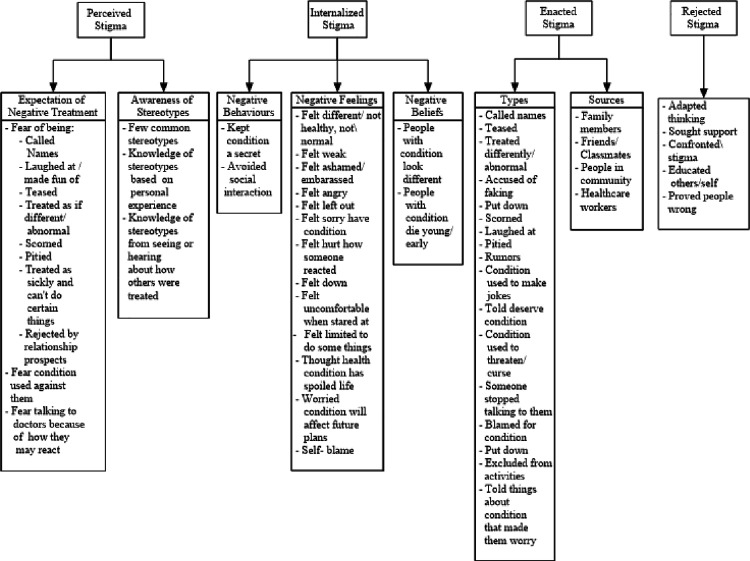
Individual-level stigma experienced by adolescents: Themes, sub-themes, categories/elements common across health conditions.

The stigma codes and categories that were common across conditions are presented according to the following stigma themes and sub-themes resulting from the analysis: Perceived stigma – Expectation of negative treatment, and Awareness of negative stereotypes; Internalized stigma – Negative behaviours, Negative feelings, and Negative beliefs; Enacted stigma – Types of negative treatment and Sources of negative treatment; and Rejected stigma. Selected quotations that are representative of the codes and categories, and sub-themes and themes are included throughout the paper. As a number of the adolescents responded to the interview questions using the Jamaican dialect, the original dialect is presented alongside a standard English translation in the results. This approach ensures that the authenticity and truthfulness of the adolescents’ experiences are preserved (see S2 Table. Jamaican dialect and English translations). Fig 1 provides a visual representation of the categories, sub-themes and themes.

### Perceived stigma: Fear/Expectation of negative treatment

Participants’ accounts across conditions were centered on various negative reactions or treatments they feared receiving if others found out or knew about their conditions. Participants feared what others would say or do to them, or what they would think about them if they knew about their conditions. Some of the negative treatments that they feared experiencing included those who knew about their conditions telling others who would then treat them badly; being called names; being laughed at or made fun of; being teased; treated as if different or abnormal; scorned; pitied; their condition being used against them; and being seen as sickly and unable to do certain things.

Participants’ fear of the various negative treatments was due to them either having experienced such treatments in the past and not wanting future occurrences, or having seen people with their conditions or other conditions being negatively treated, or having heard negative things said about these people and not wanting the same thing to happen to them. Sample quotations from the transcripts are presented below.

Here, participants across health conditions did not want to be scorned, pitied or seen as sick/sickly:

Most people are not knowledgeable about diabetes; so, is like they would hear and then seh [say], “Oh, you have suga [sugar], my grandmadda [grandmother] have it, one bag a summen [something] dem [them] tek [take].”…Yeah, so, they just don’t understand. So, I was afraid of people scorning me and don’t want to talk to mi [*me*] …

*(Female 1, 15 years old, Endocrine condition – Diabetes)*

Because some people don’t understand, so they give you mixed emotions sometimes when you tell them, right? You know, scornful or pitiful. I don’t like any of the two emotions, so I keep it [a secret].

*(Female, 15 years old, Sickle Cell Disease)*

And, when I am taking dem [them] at school…, nobody don’t see me taking dem [them], Miss. Because I don’t want anybody to know that I take pills…. because dem [they] a [are] guh [going to] seh[say], “Sick people alone tek [take] pill or mad people.”

(*Male, 16 years old, Mental illness*)

Here, participants across conditions described not wanting to be made fun of after having seen it happen to others:

Because (sighs) I just don’t want everybody to know about mi [*me*].... Well, some of the kids, like….didn’t trouble me, but I just don’t want everybody to know... I don’t really know how to explain it.... I think some of the kids in my class now will make fun of me…

(*Female, 13 years old, Endocrine condition - Overweight*)

Also common across conditions was a fear among participants that others knowing about their condition or them disclosing their health condition to others would lead to them being rejected by their relationship prospects. Older participants, in particular, females, appeared to have this fear.

[The guy I like], I don’t want to tell him that because him nah [not] guh [going to] like mi [me] if mi [I] have that...... Him [He] a [is] guh [going to] seh [say] him want him girl fi [to] be nice and sweet.

*(Female, 19 years old, Mental illness)*

I have a girlfriend now; she doesn’t know, though...... Because is like…..I don’t want to stress her yet. ….. I want[ed] to tell her when… I saw her the other day, but I didn’t…. disclose it….. I guess one of the reasons why I haven’t…. when I tell her, that will really solidify whether or not that you want to be in a relationship with me or not.

*(Male, 18 years old, HIV)*

Like, I tell my mummy not to tell persons, like boys, that I have this disease. But of course, she does tell them.… I just don’t want them to know... I just don’t like telling them that I have [a] disease...... I will tell him eventually…...when I get a ring.

(*Female, 17 years old, Sickle cell disease*)

Although not continuously explored with all the conditions, a few participants across conditions, in particular males, seemed to have had a fear of talking to their doctors or asking questions regarding concerns they had about their conditions because of the reactions they might get from them.

[I don’t really tell the doctors about concerns I have about my illness], because sometimes the doctor says at my age [I should] know these things. So, I kinda [kind of] feel embarrassed to ask them some of these questions.

*(Male, 14 years old, Sickle cell disease)*

You see, you [need to] understand it that if [I] ever told the doctor this, [it would be] like being in a next lab down here being admitted again… I fear that.

*(Male, 18 years old, Mental illness – depression)*

### Perceived stigma: Awareness of negative stereotypes

Participants across health conditions, in particular older adolescents, articulated only a few negative stereotypes regarding people with their specific condition, and this was usually based on experiences they had personally, in the past. A few of them also became aware of stereotypes from seeing or hearing about how others responded regarding people with their conditions or other conditions. Some participants also internalized these stereotypes regarding people with their specific illness, as these manifested as various negative beliefs about themselves or elements of internalized stigma which are described later in this work. Here are examples of quotations from across conditions suggesting participants’ awareness of societal stereotypes regarding their conditions:

…people say that I am not going to make it, I am just going to make it to sixteen…. People at school, adults, I hear them tell other people.

*(Male, 12 years old, Sickle cell disease)*

[I got tested for HIV routinely] because I was fearful, because I was always fearful as a child. I would say I was always fearful [about] what I [saw] coming …. on the television. …..... I guess then I had the stereotype in my head that people of colour are normally…. HIV positive... That is how it was……that is what was portrayed..... like on the media, you know, like people from Africa, and then I know I am of African descent...

(*Male, 18 years old, HIV)*

Everything was like it was in a jail or something.... And the problems that you have and lack of communication, because you thought that the other person on this ward, being what it was to the outside public…. a mad ward*...* That’s what I heard people say. That’s what people say and that’s what deejays dj about, …. Ward 21. And if anyone really knows that I was admitted at Ward 21, I would be a total disgrace or I would be classified as a madman.

(*Male, 18 years old, Mental illness – Depression)*

It was really, you know ….. like rarely for me there [are] some comments thrown out there, probably not meaning to hurt…but they just for some reason hurt me. Like the fact when some people are talking about how much weight people have on…and constant judging, and they talk about people that probably most of the times fall into the range of my size.

(*Female, 14 years old, Endocrine condition – Weight issues & Early cycle)*

### Internalized stigma: Negative behaviours

All the participants across health conditions reported that others knew of or were told about their conditions besides their parents and doctors. However, for the majority of participants, the people who knew varied as they felt it was necessary that either their conditions or that they were taking medication, or attending clinic be kept secret from some people due to a fear or an anticipation of what people would think, say or do. Some participants initially indicated that not disclosing their conditions to some people was for privacy reasons in that they did not want people to know their business. However, when they were questioned further during the interviews, it was noted that the secrecy about their conditions was driven by a fear of being negatively treated rather than for privacy. Some participants also specified how they ensured that others did not find out about their condition. These included not volunteering information when the topic was being discussed, withholding or lying about the cause when unwell, pretending to be okay when sick, and hiding their medication. Below are some examples from the transcripts:

I don’t really tend to like, “Oh gosh, I am feeling sick!” I like, keep it to myself and like try to pretend as if I am … ok until I guess I reach home when I can throw all the tantrum I want and cry…

(*Female, 18 years old, Sickle cell disease*)

Miss, [when I have to take my meds at school], I just go at di[*the*] pipe…. the private pipe….and tek [*take*] it Miss.

(*Male, 16 years old, Mental illness*)

A few participants even expressed regret at some point that specific people knew about their conditions because of the negative reactions they had gotten from them.

One boy in [my] class – [I wish he didn’t know that I have sickle cell].... He would just shout it out every minute him talking to people……and tell everybody and make pure joke bout [about] it. That was in [Grade 7], though.

(*Male, 12 years old, Sickle Cell Disease*)

And then going back to [the] friend that I told you that I regretted I told.... Well, in 4^th^ form I told my friend, and she went and told another person. The two of us were talking, but we weren’t that close. And so, I didn’t really want her to know. So, … she had a drink …… and wi [we] normally share ….. our food and drink, and stuff. So… I was like, “Can I get some of your juice?” She was like, “No, you have Diabetes!” ….. like the way she said it, made me feel uncomfortable. ……. it wasn’t like it was just us there, there were a lot of persons around. ……..

(*Female, 17 years old, Endocrine condition – Diabetes*)

Participants across conditions also commonly indicated that they avoided social interaction with those whom they perceived treated them negatively or would treat them negatively. In some cases, the avoidance of social interaction with others was temporary, while for other participants, this was permanent.

Yeah, …… like the people on my mummy’s side, they don’t understand. So…they are on my case…. whenever they come or they call, I just…isolate myself… because I am tired of hearing them say, “You can’t eat that. You must eat that.”..…And sometimes they would scold me about the weight. …… And it’s kind of annoying. So, that’s why I isolate myself*.*

*(Female, 15 years old, Endocrine condition – Hormonal issues & Overweight)*

I couldn’t do anything at the time [when they said I was faking] because I was having really bad chest pains. …. But, like when I got back to them, …..I asked them if they were crazy….. And …., we kinda [kind of] didn’t talk for a while because I [was] like, “How can you say that to someone when you have never been in their position? You don’t know what it is like to kinda [kind of] just stop breathing.” And…. the topic came up again, and they kinda [kind of] like said sorry. They know [what] it is all about now, and it’s kinda [kind of] serious.

(*Female, 15 years old, Pulmonary condition – Asthma*)

Yeah, [I have lost friends], because whenever time a friend trouble mi [*me*] about it, that’s when I drop their company.… [I have dropped] even big people who, me a dem [*them*] friend.

*(Female, 18 years old, HIV)*

For a few participants, the negative reactions from others seemed to have been so overwhelming and difficult to handle that they went to great lengths to avoid interaction with them. For example, here, Linda (not her real name), an 18-year-old female with HIV described dropping out of school and leaving her community for a while because of negative treatment:

Dem [They] will seh [say] “…..Yuh [You] have AIDS fi [for] true? Mi [I] nuh [don’t] want yuh [you] come a [to] mi [my] seat.” Or they will scorn, and trouble yuh [you] and seh [say], “Mi [I] nuh [don’t] want yuh [you] really too eat from the spoon or drink nothing and mek [make] it even guh [go] near my bottle or whatever.”.... So, one morning mi [me] just get up and seh [say] to mi [me] boyfriend seh [say] …… “mi [me] nah [not] guh [going] back a [to] school.”.... Him seh [say],“Ok, I [will] get you into a school where dem [them] don’t really know much about it.”...... Anyway, mi [me] never too go out with it. Mi [me] just stay [in] fi [for] round two years, seh [say] mi [me] nah [not] guh [going to] school, and just stay in, clean him house and whatever.

Here, another female, Elaine (not her real name), 17 years old with sickle cell disease, described changing school because of negative treatment and non-supportive teachers:

Like… this girl, she went around spreading that I was pregnant because …the gallstones that I have made my belly look puffy. So, everyone believed and started saying that I was pregnant. But I was not. She was the one who was pregnant. ….. So, that went [on] all throughout [being at my previous school] … and now it is starting to spread at [my present school, and I don’t know how it got [there].

### Internalized stigma: Negative feelings

Participants across health conditions described a number of negative feelings they experienced that were due to the stigma of their conditions. The most common negative feelings across conditions were participants’ feelings of being different from their peers and others, and not as normal.

‥ my mum always tells me that I am normal; I am the same as them. But …. I don’t think I am normal because my red blood cells are not normal; they are not shaped the right way. That’s why I think I am not normal….

(*Female, 14 years old, Sickle Cell Disease*).

Yes… Dem [*them*] normal…. because dem [*them*] nuh [*don’t*] affi [*have to*] tek [*take*] nuh [*no*] insulin.

*(Male, 13 years old, Endocrine condition – Diabetes)*

Despite feeling different from others, though, due to their conditions, a number of the participants did not want to be seen or treated that way. Hence, they employed various strategies of secrecy, highlighted earlier, to limit peers’ and others’ knowledge of their conditions, so that they could pass or be seen as normal.

Participants across conditions also commonly described feeling limited in doing certain things due to their conditions. Female participants mainly expressed this:

I don’t really have that many problems, but it’s just...I can’t do things that normal children can do. That is basically it, I guess.

(*Female, 14 years old, Sickle Cell Disease*)

Yes.... I always thought that I was weak, and I couldn’t move most things.... And that I can’t do [things] that the others do.

(*Female, 13 years old, Pulmonary condition – Asthma)*

It affect me, like, it come in like I am abandoned... Because I can’t do certain things [like others can].

*(Female, 18 years old, Neurological condition – Epilepsy)*

However, sometimes this feeling was not based on any observable limitations from their conditions, but due to restrictions likely placed on them by their parents or others, which they had come to accept or internalized. Sometimes, however, the participants tried to resist this:

But when my mother always use this term like, “Yuh [You] have to rememba [remember] seh [say] yuh [you] nuh [not] normal like di [the] rest a [of] wi [us].” … Certain things she like restrict my action. Like when mi [me] little, shi [she] monitor how long mi [me] stay in the wata [water] and those stuff. And being me, I am always determined to lead a normal lifestyle. So, I really don’t like that part about “You rememba [remember] that yuh [you] different.” Mi [I] nuh [don’t] really find miself [myself] different… (hisses teeth).

(*Female, 18 years old, Sickle Cell Disease*)

I hate being treated as if I can’t do anything by my dad, … and my aunt. Because they will be like, “You’re epileptic. You can’t do things.” Like, yuh [you] know, I am a teen; so, I am interested in parties and all of that……I see my cousins go out and everything, and I am like, “Why can’t I go out, too?”*….*I am always saying to my dad, “You’re not always going to be there for me; you have to allow me to grow up.”

*(Female, 15 years old, Neurological condition – Epilepsy)*

Other negative feelings that were commonly experienced across conditions include angry/anger, regret/felt sorry have condition, felt embarrassed, felt left out, felt/thought health condition has spoiled life, worried condition would affect plans for the future, felt down, felt hurt by the way someone reacted, felt uncomfortable when stared at, and self-blame. Table 2 provides illustrative examples of each of these negative feelings.

**Table 2:** Sample illustrative quotations of adolescents’ experience of negative feelings.

Type of negative feeling	Illustrative quotations across health conditions
**Angry/ Anger**	[At school, the children tease, say mean things,] …pick fights, but I walk away…I have a temper, so at times I will fight, not proud to say… I get counselling for it, and when I fight, that’s how I express my anger. (*Female, 15 years old, Neurological condition - Epilepsy*) Some honestly don’t know; they think I have asthma or something else. My little cousin was talking to a next little cousin of his, and I was walking, and I hear him say, “Him have disease, yuh [*you*] nuh [*know*]?” I confront him – he is younger than me – and I confronted him, and he said he never said that, and I was right dere [*there*].…. I was just sort of angry about it. [Him] telling people that I… have disease like that…Like him want dem [*them*] fi [*to*] scorn mi [*me*] or someting [something]. (*Male, 12 years old, Sickle cell disease)*
**Felt left out**	Well, I get mad sometimes because I know I can’t do things that normal people can do… So, I feel abnormal, and when I see my brothers going out, yes, I am going to feel left out. So, what I normally do is, I just go in my room, cry about it, and then immediately I will get over it. (*Female, 15 years old, Sickle cell disease*). I would see other children eating ice cream, cake, and those stuff, and I am a child, I love those stuff. So, yes, I would like, feel left out and all, but mummy… kind of helps me out because.... she [would] buy the ice cream, but it [had] to be in moderation. (*Female, 12 years old, Endocrine condition – Diabetes*)
**Regret/ Felt sorry have condition**	Well…I feel comfortable around everybody …. but sometime I do regret having it … Because I didn’t want to have any disease or sickness, I just didn’t want to have any. (*Female, 15 years old, Endocrine condition – Hypothyroidism & Overweight*) Dem [them] nuh [not] better dan [than] mi [me]. Even though sometimes whenever time mi [me] si [see] dem [them], mi [me] woulda [would] seh [say], “Buoy, mi [me] wish mi [me] was like dem [them] nuh [don’t] have dah [this] sickness yah [here]...” (*Female, 18 years old, HIV*)
**Felt ashamed/embarrassed**	[I have felt embarrassed about having my condition] one time in Grade 8 when I had to explain it. In Grade 8….. my teacher, she was talking about [having a health condition]. So she asked if anybody [has it], she seh [said] wi [we] fi [are to] just put up our hand if wi [we] have it. And when I put up my hand, she said that I must explain it, but I didn’t want to. …. I told her… And when I told her I didn’t really want to do it ….. she seh [said] just feel comfortable and explain it. (*Female, 15 years old, Endocrine condition – Hypothyroidism & Overweight*) Yeah, [I have felt embarrassed about having my health condition…. Because… I think people woulda [would have] take [taken] it just normal [when them find out]... [Instead], dem [them] si [see] yuh [you] and trouble yuh [you]. It mek [make] yuh [you] feel like a way...knowing that you have it and dem [they] a [are] guh [going to] si [see] yuh and everyday dem [them] rubbing it in your face. That mek [make] you feel bad. But worst is dem [them] si [see] yuh [you] get mawga down [meager], that mek [make] it worse. And dem [them] nuh [don’t] si [see] seh [that] a [is] dem [them] a mek [make] yuh [you] get mawga down [skinny]…. (*Female, 18 years old, HIV*)
**Thought health condition has spoiled life**	Yeah, it mash up my life badly. Because if, alright, if mi [me] si [see] dis [this] slippers and mi [I] seh [say], “mummy mi [I] like dat [that].” [She would say,] “No, yuh [you] cyaan [cannot] wear dat [that] because di [the] bottom a [of] it too tenda [tender]. Summen [Something] a [is] guh [going to] jook [stick] up inna [into] yuh [your] foot”..… (*Female 1, 15 years old, Endocrine condition – Diabetes*) Because if my boyfriend should say he want to go or whatever, I would be fretting because who is going to accept me with this sickness? There is nobody out there… [who would] accept you just like that, because I [have] been there… (*Female, 18 years old, HIV*)
**Worried condition will affect plans for future**	I will probably only get to do some of the things I want to..… such as …. I know I want to do research, and I know I wanted to …. become a doctor in America or something. You know, people like the workplaces there …… And [with] HIV …, and you are an international student……, when you go in, you have to do blood work…... People might say, … “We can’t, insur[e]….,” don’t want to cover [you]…. so…some stuff might not happen. (*Male, 18 years old, HIV*) One of my friend[s], …. she said to me one day, …“So, you know that persons with Sickle Cell is, when they reach fifty, they are going to die?” …Mi [I], did really feel a way like. So, mi [I] seh [said], “So, why yuh [you] a tell me dis [this]?”... I really want to, well achieve a lot of things before. And when I think about all the years of schooling... I am kinda [kind of] like “….. half a mi [my] life gone! And I have [until] about fifty?!” ….. I am thinking about it... like what I want to do. Like one time I want to become a lawyer, but then I am looking at it. Like, it’s going to take about twenty years ….in the business before I actually start, like… mi [I] nuh [don’t] know... (*Female, 18 years old, Sickle cell disease*)
**Self-blame**	yeah…. [I blame myself]. ‘Cause I got it when I was small, and I used to have a lot of sweet things. Every day, I would have continuous sweet things and in large amounts. I think I was being greedy; so, that’s why I blame myself…. because I think they said that I overworked my pancreas, yeah… (*Female, 15 years old, Endocrine condition – Diabetes*) I do [blame myself]. But I guess partial blame, not everything…. I just wish I hadn’t like…I was… a sheltered person. I was really sheltered. I didn’t go…. I don’t even really like to communicate with people much. So, I don’t know how I got up that time and just go have sex with somebody. So, it’s like I am saying, “what the hell happened this time? That, that one time, that one single time.” (*Male, 18 years old, HIV*)
**Felt hurt by the way someone reacted**	Like, sometime my friends at school would run jokes, like [when] mi [I am] sick, but mi [I] know seh [that] dem [them] a run joke, but sometimes it kinda [kind of] hurts….like, in 9^th^ grade, wi [we] use to have days, like, one day somebody get troubled, a next day dem [them] trouble somebody else…when a day come and dem[them] seh [say] a [it’s] my day and dem [them] trouble me…mi [I] know seh [that] a [is] joke dem [they] a [are] mek [making] …[but] mi kinda [kind of] feel a way, yes. (*Male, 16 years old, Sickle cell disease*) …. when they trouble me … It hurt mi [me] …. but it nuh [don’t] hurt me that tears come a [to] mi [my] eye or whatever. Mi [I] just hope that one day mi [I] coulda [could] get cured so that mi [I] can show dem [them] mi [my] paper *say*, “Look here, mi [I] nuh [don’t] have nuh [no] HIV.” (*Female, 18 years old, HIV*)
**Felt down**	Well, sometimes I wish the symptoms didn’t affect me most of the time, but I think of it [as] kind of a negative part of my life……And I kind of feel down about myself. (*Male, 13 years old, Sickle cell disease*)
**Felt uncomfortable when stared at**	Because I don’t like when I am in the doctor’s office, and they let me blow the paper to see what’s wrong with me and everybody is just in the room. I don’t like when people stare at me.... It just makes me feel weird.... abnormal.... Because when they, I am doing it and everybody is just staring at me…I just, [a] thought come in my head, “So I wonder what they are thinking about?” (*Female, 14 years old, Neurological condition – Epilepsy*) Yes, anytime people see me having it… People see me, they looking at me and those stuff, I don’t like it…. I’m like that ‘cause sometimes I do things for myself, and people want to help me and those stuff and I don’t like it…. I just like when people do not notice a lot about me. (*Male, 15 years old, Neurological condition – Epilepsy*)

Some participants’ experience of the various negative feelings was not ongoing; however, their experience might have been one-off, or it changed over the course of their lives with their conditions, where it was resolved through cognitive maturity or development, or through education. Below are examples from the transcripts.

A female participant shared that she believed at one point she caused her diabetes until she researched it:

[My family], they mek [make] it look like I cause it on myself. Most of them cuss [curse] mi [me] and tell mi [me] a lot of things, like I eat too much sweets …… Like, one point, … [I believed what they said]... until I start researching for myself about diabetes.... And I found out that it’s not my fault.

(*Female, 18 years old, Endocrine condition -Diabetes*)

A male participant with HIV described that he thought he would not be able to achieve his plans and goals:

Well, [when I found out] …. that was …. one of the most defining moments in my life, because everything just started to flash in my head, I think… and I started to sweat. My head started hurting. But I tried to keep my composure because I had to walk out of the room, you know, where …everybody was standing.... I was just thinking about, you know, how much plans and things I wanted to do and stuff. How much, you know, goals I have set and stuff. And I just felt like this just wiped everything away. Of course I cried..... it was just a sad state of affairs.... I was just fretting. I didn’t know what to do, really. And then I found out a little bit after that I was going away on the …. scholarship …. I even felt like if I didn’t press for that, I wouldn’t have gone, because then I felt like I couldn’t even enter the United States with being HIV Positive… [but then]... I didn’t have to...[disclose].

*(Male, 18 years old, HIV)*

### Internalized stigma: Negative beliefs

A number of participants across the conditions internalized stereotypical beliefs pertaining to people with their conditions. These manifested as negative feelings and concerns about self. For instance, a number of them expressed concern about aspects of their appearance. This seemed to have been partly based on them having internalized the negative stereotype regarding how people with their conditions looked:

Well, because of my weight …. I have …. low self-esteem. It’s kind of in the middle.… especially when I have to do swimming...So, sometimes I don’t want to do swimming because I am like, “I don’t have the swimsuit body.”

(*Female, 15 years old, Endocrine condition – Weight issues & Hormone imbalances)*

I don’t like taking off my clothes in public because, like, I am really skinny……. Like, that is the other downfall to this disease. Like, persons with sickle cell tend to mature less, not as fast as the average child. I would like to kinda [kind of] get fattish and…

(*Female, 18 years old, Sickle Cell Disease*)

Some of them, however, were of the view that they did not look the way people with their condition typically look, and so, someone would not be able to tell that they had their illness.

[I prevent the other students in my class from finding out by] … I just don’t tell them. And because I look normal, they just don’t suspect anything.

(*Female 2, 15 years old, Endocrine condition – Diabetes*)

No Miss, [I don’t think that somebody can look at me and tell that I have sickle cell]. Mummy said I am very fat for someone who has Sickle Cell. So, I don’t look like I have it because I look healthy. [For somebody to look at somebody and tell that they have Sickle Cell], I think that they would be slim….

(*Female, 13 years old, Sickle Cell Disease*)

Another example, females across conditions were concerned about their specific condition getting worse or dying from the condition. This seemed to have been based on their belief in the stereotype that people with their condition do not live long or die early.

One of my friend[s], …. she said to me one day ….. “So, you know that persons with Sickle Cell, is when they reach fifty they are going to die?”… Mi [I] did really feel a way like. So, mi [I] seh [*said*], “So, why yuh [you] a tell me dis [this]?” *….* I really want to, well, achieve a lot of things before. And when I think about all the years of schooling...I am kinda [kind of] like, “…. half a [of] mi [my] life gone!”

(*Female, 18 years old, Sickle Cell Disease*)

Well, [I have seen and heard that HIV] make you get sick…and make you get slim… and…. it don’t make you have last long life. It depends on if you take your medication. [It bothers me] sometimes, because mi [me] nuh [don’t] waan [want to] dead.

*(Female, 16 years old, HIV)*

### Enacted Stigma: Types of negative treatment

The majority of participants across all the conditions described having experienced some form of negative treatment from others due to their conditions. Some negative treatments were overt while others were subtle. Being called names was the most common negative treatment participants reported experiencing. Some participants, however, reported that they were not continuously bothered by this. This was due to a change over time in their interpretation of the treatment as negative and offensive because of information they had received, which caused them to re-label their experience, seeing the treatment as justified or excusable.

…. now in high school everybody have a nickname. Me, mi[me] a Crutchy because mi [me] used to walk wid [with] a crutch stick.... that was when I was in …. 8^th^ Grade …. when I found out I have AVN [avascular necrosis]. I had to walk with the crutch stick, and I was like, “I don’t want [to] walk wid [with] it come to school. ….. But [now] when dey [they] call mi [me]... a nickname, … suh [so] everybody has a nickname, suh [so] wi [we] just laugh and gwaan [go on]. Cah [Because], now dem [them] si [see] mi [me] and a hail mi [me]. [Either] dem [them] a seh [say], “Crutchy,” or …..dem [them] a seh [say]… mi [my] last name..

(*Male, 14 years old, Sickle Cell Disease*)

But some of them don’t … understand why dem [them] call mi [me] sugar, because dem [them] call mi [me] Sugar Man at school.... but mi [me] nuh [don’t] have nuh [no] problem wid [with] it. When mi [me] did young, mi [me] use to fight fi [for] [*when*] people call mi [me] suh [*so*]. But since mi[me] get older and start guh [*go*] a camp…mi [me] understand a just nickname …. mi [me] understand seh [*say*] a just life. Everything happen fi [*f*or] a reason in life. Suh [So], yuh [you] cyaan [cannot] blame life…Is a reason why…. God mek [make] mi [me] have Diabetes…

(*Male, 16 years old, Endocrine condition – Diabetes)*

Other negative treatments that the participants reported experiencing that were common across conditions included condition being used to make jokes; condition being used against them to curse them and to threaten them; someone stopped talking to them; told deserved condition; gossiped about or rumours spread about them; pitied; laughed at; scorned – (others did not want to touch them, take things from them, or acted like they were contagious or scorned them); excluded from groups and other activities – (other children kept them out of their groups and games, or didn’t want to participate in groups with them); blamed for condition; put down – (people made critical/judgmental comments about them); teased; treated differently or as if abnormal; told things about their condition to make them worry; and accused of faking or using condition as an excuse. Table 3 below provides illustrative quotes for each type of negative treatment.

**Table 3:** Sample illustrative quotations of adolescents’ experience of other acts of enacted stigma.

Type of negative treatment	Illustrative quotations across health conditions
**Condition used to make jokes**	Like, sometime my friends at school would run jokes, like [when] mi [I am] sick. but mi [me] know seh [say] dem them] a run joke, but sometimes it kinda [kind of] hurts…. (*Male, 16 years old, Sickle cell disease*)
**Laughed at**	Yeah, one of the time when I was wetting the bed…and my sister and bredda [brother] dem [them] neva [never] understand. So, like, they laughed at me. (*Female, 15 years old, Sickle cell disease*) Deh [they] call mi [me] Sickle Cell, deh [they] are laughing and deh [they] told mi [me] said it’s good for mi [me] that I ave [have] Sickle Cell. (*Male, 13 years old, Sickle cell disease*)
**Rumours spread about them**	Alright then, how I cover it up now, uhm, by people like they just heard rumours. But I don’t know, but I just cover it up most times. …. It was like they were saying that I was half crazy… That mi [me] did have a pickney [child]…. (*Male, 18 years old, Mental Illness – Bipolar*) Like….. this girl, she went around spreading that I was pregnant because …. the gallstones that I have made my belly look puffy. So, everyone believed and started saying that I was pregnant. … So that went [on] all throughout [being at my previous school]… and now it is starting to spread at [my present school] (*Female, 17 years old, Sickle cell disease*)
**Pitied**	Well, it was in Grade 7, and our Religious Education teacher asked us if you have any illnesses and do you think that God will help you and all of that. So, when she asked everybody, most of them said, Asthma. And when I said Sickle Cell, a lot of people were like, “Really? Sickle Cell? Oh my God, I feel sorry for you!” and all of that. And so, it kind of went out. (*Female, 15 years old, Sickle cell disease*) Well, they [my brothers and sisters] feel sorry for me, but they [also] trouble me...call me seizure boy and one bag a [of] summen [*something*]. (*Male, 18 years old, Neurological condition – Epilepsy*)
**Told it’s good to have/ Told I deserve condition**	Deh [they] call mi [me] sickle cell, deh [they] are laughing and deh [they] told mi [me] said it’s good for mi [me] that I ave [have] sickle cell. (*Male, 13 years old, Sickle cell disease*) Yes, my brother, when he is upset with me, because we fight, and he will use the Diabetes sometimes. Like, if he has something put up for himself, something sweet, and I eat it, he will get upset and complain and quarrel, and say I am not supposed to be eating it and I am craven and ….that’s why I deserve the Diabetes (*Female 2, 15 years old, Endocrine condition – Diabetes*)
**Condition used to curse them**	Dem [Them] [my brothers] cuss [curse] me, and nuff [a lot of] time dem [them] trouble mi [me] fi [for] mi [my] tings [things]. Suh [So], mi [I] cuss [curse] dem [them] fi [for] mi [my] tings [things]. Dem [them] tell mi [mi] tings [things] sometimes … like mi [me] have…mi [me] sickness …. dem [them] tell mi [me] mi [me] have AIDS and dem [them] cuss [curse] mi [me]. (*Female, 14 years old, HIV*)
**Condition used to threaten**	Like mi [my] teacher dem [them], mi [me] really hate [this], but like right now, it nuh [doesn’t] matter, mi [I] just laugh it off. ….. Like I [am] talking to my friends in the class or summen [something] …dem [them] a seh [say], “Memba [Remember] mi [I] have Sickle Cell, one lick mi [me] bruk up [break],” or something like dat [that]. And mi [me] a seh [say], “A [Is] wha [what] dat [that]?” Dem [them] a seh [say] like mi [me] fragile. (*Male, 14 years old, Sickle cell disease*) One day a boy said, he come inna [into] the line and bore [push in front of] me. [Before], him neva [never] did know seh [say] mi [I] have seizure …. but now him know seh [say]… mi [me] have seizure, him a trouble mi [me] and him a seh [say], tru [because] when I told him …, “Why did he bore [push in front of] me?” And him a seh [say] I must “stop the noise before him hit mi [me] in mi [my] head and I ketch [catch] seizure.” (*Male, 15 years old, Neurological condition – Epilepsy*)
**Someone stopped talking to me**	I was talking to somebody……and I tell [him] that I have it, and he stopped talk to mi.[me] (*Female, 14 years old, HIV*)
**Scorned**	Dem [Them] will seh [*say*], “… Yuh [You] have AIDS fi [for] true? Mi [I] nuh [don’t] want yuh [you] come a [to] mi [my] seat.” Or, they will scorn, and trouble yuh [you] and seh [*say*], “Mi [I] nuh [don’t] want yuh [you] really to eat from the spoon or drink nothing and mek [make] it even guh [go] near my bottle or whatever.... (*Female, 18 years old, HIV*) They would call me ‘sugar mama,’ ‘sugar belly’ I can’t really remember the rest of them… you have few who scorn me, like they don’t ask me for anything and they keep a distance from me…. I would tell them that it is not contagious, and you can’t get diabetes by touching the person or going near them. (*Female, 18 years old, Endocrine condition – Diabetes*)
**Blamed for condition**	They make it look like I cause it on myself. Most of them cuss [curse] me and tell me a lot of things, like I eat too much sweets and most of them…never helped… (*Female, 18 years old, Endocrine condition -Diabetes*)
**Put down**	What [the doctor] was saying [was] that …. my weight [was] ok…. So ….. I walked out [of the clinic] with my spirits raised because I was pretty put down [earlier]. I don’t even know why I was put down so far, and normally, I am not. [It’s] because I was like going because of what the nurse [had] said. I was going there [to the clinic] expecting to be like impressed because I have been working hard… since September. Even in high spirits, saying, “Ok, I have taken off a lot of weight. I am going to be let off clinic today.” No[w], only to have the nurse, like, completely put me down. (*Female, 14 years old, Endocrine condition – Weight issue & Early cycle*) Knowing that people still, like, put me down, constantly put me down, but I am getting over it… (*Female, 18 years old, Mental Illness – Bipolar*)
**Teased**	Well, they used to tease me about my weight, but not in High school…. I mean, in High School, ….. I get to meet people … who weigh more than me…by far.. (*Female, 15 years old, Endocrine condition – Weight issues & Hormone imbalances*) People …call mi [me] sickle cell… and a tease mi [me] seh [say] mi [me] ave [have] sickle cell. ….. some…. classmates at [my previous school], but dey [they] graduate (*Male, 13 years old, Sickle cell disease*)
**Treated differently or as if abnormal**	… she [my classmate] had a drink...and wi [we] normally share … our foods and drink and stuff. So…. I was like, “Can I get some of your juice?” She was like, “No, you have Diabetes!” …. like the way she said it, made me feel uncomfortable …. it wasn’t like it was just us there, there were a lot of persons around and so that made me feel uncomfortable… And so, from then I just decided that people that know, treat you differently, when in her case, she treated me differently from the rest of the persons. (*Female, 17 years old, Endocrine condition – Diabetes* So here I am now trying to find a way to tell him …. and then I said, “If I tell you something, will it change your opinion about me?” So, he said, “No.” So, I typed the message really fast, and I sent it immediately, and turned off my Wi-Fi, and I turned off my phone. So, I am like, “I don’t want to face it right now because I don’t want to see his reaction.” Mi [I] si [see] him number on my phone and he is like, “Really? Ah, explain to me what it is.” And I explained to him. And so, he was like, “So, that’s sad.” So, I [said], “Yeah, I know, but I don’t like when people pity me or when people treat me differently,” and from ever since we have been really close. (*Female, 15 years old, Sickle cell disease*)
**Accused of faking or using condition as an excuse**	Well, sometimes persons in my school think that when I have an attack, they sometimes think I am faking. But then, that kind of hurts me because they know the person I am, I am not like that. (*Female, 15 years old, Pulmonary condition – Asthma*) Well, it all happened when I was at PE [physical education], and my knees and my ankles started to really hurt. So, I told the PE [physical education] teacher and ….. I got to sit out the class…. And because we are doing a course that nobody liked.... everybody was saying, “Oh, is just because…. she have sickle cell and she faking and all of that stuff.” And I am like, “Why would I fake it if it is something real?” (*Female, 15 years old, Sickle cell disease*)
**Excluded from groups and other activities**	Sometimes they wouldn’t want to put me… like, Miss say, “Choose your group.” Sometime they wouldn’t want to put me in their group…and games as well. (*Female, 12 years old, Endocrine condition – Diabetes*) So, that is why I repeated, because I couldn’t manage the first half that I was with. Dem [them]call mi [me] names, and they didn’t want to participate with me in groups and… Draw away from me when I am in class. (*Female, 19 years old, Mental Illness – Bipolar*)
**Told things about condition that made you worry**	I don’t like when they trouble me about it.... They seh [say] they going to video mi [me] when it [a seizure] is ketching [catching] mi [me] …. But I just don’t like it…. and mek [make] when mi [I] come back to miself [myself], mi [I] watch it and si [see] how mi [I]... react… It keep bothering mi [me].... Every time mi [me] have it on mi [my] head that dem [them] going like trouble mi [me], video mi [me], then mi [I] a [am] guh [going to] watch miself [myself] and a guh [going to] si [see] how mi [I] look bad... (*Female, 18 years old, Neurological condition – Epilepsy*)

Some participants’ experience of the various negative treatments, however, was not ongoing or continuous over the course of their conditions. The experience was either one-off, or it changed as the youth got older, changed school levels (i.e., moving from primary to secondary) and peer groups, or as they took steps such as non-disclosure/ secrecy.

Well, they used to tease me about my weight, but not in High school…..I mean, in High school …. I get to meet people …… who weigh more than me…by far; so, they don’t tease me about my weight in High school…. According to them, I am not fat. I am thick and thick is the new thing that’s in right now…

(*Female, 15 years old, Endocrine condition – Weight issues & Hormone imbalances*)

People …call mi [me] sickle cell… and a tease mi [me] seh [say] mi [me] ave [have] sickle cell. ….. some…. classmates at [my previous school], but dey [they] graduate, dey [they] call mi [me] sickle cell, dey [they] are laughing and dey [they] told mi [me] said it’s good for mi [me] that I ave [have] Sickle Cell.

(*Male, 13 years old, Sickle cell disease*)

A sense of being negatively treated was also experienced by a number of participants across conditions who reported that they were treated as if they couldn’t do certain things. This was interpreted by some of them as being treated softly or like a baby, which they did not like:

…and some of them would like babying me and like don’t want me to do certain stuff... Because I want to do certain things and they are behaving like I can’t do nothing at all. If I want to do something for myself, they will tell me, “No, I will do it.” And like I have to just sit down and watch.

(*Female, 16 years old, Sickle Cell Disease*)

Ok, number one, I hate being treated as if I can’t do anything by my dad…and my aunt. Because they will be like, “…. You’re epileptic. You can’t do things.” Like, yuh [you] know, I am a teen; so, I am interested in parties and all of that … I see my cousins go out and everything and I am like, “Why can’t I go out, too?”

(*Female, 15 years old, Neurological condition – Epilepsy*)

Interestingly, a few participants did not interpret being treated like a baby or softly as pejorative or negative. Instead, they seemed to have thought the opposite, in that they found the experience quite positive and welcomed the treatment. It appeared to have been more like a nurturing experience for them in that they seemed to have felt cared for.

…. All my friends treat me like I am a baby or something. And they baby me any time because they know I can’t get wet by the rain. They say, “Oh no, we can’t leave school now. We want the rain die down, so, she [don’t] walk in the rain,” …..

(*Female, 15 years old, Sickle Cell Disease*)

My mummy kinda [kind of] treat me a little soft. But my daddy’s behaviour don’t change..... I enjoy [how my mother treats me].

(*Female, 18 years old, Neurological condition – Epilepsy*)

Some participants across conditions also seemed to have felt negatively treated by seemingly well-intentioned actions of others when they would constantly check on them or monitor their behaviours or actions because of their health conditions. Though the actions may have resulted from a place of others being concerned for them, it was possible that the actions may have been motivated by a sense of blame towards the adolescents for putting themselves at risk, which some of them may have felt. And so, they (the adolescents) viewed the treatments as overprotective and found them unhelpful and excessive.

Well, [my family members], they pretty much are overprotective. …..Like, if I am going outside, they always want me to put on a jacket. If the time is cold, they want me to put on socks and jacket, and stuff like that.

(*Female, 13 years old, Sickle Cell Disease*)

Well, my aunty on my father’s side, she is very overprotective. So… like, she lives overseas, but when she come down and I have to spend time with her, I always prepare myself. Like, alright, I spend with her, like three weeks. [So], one whole three weeks of controlling my diabetes. When I come back to mi [my] madda [mother] now, yeah, back to mi [my] normal lifestyle.

(*Female 1, 15 years old, Endocrine condition – Diabetes*)

### Enacted Stigma: Sources of negative treatment

The various negative treatments the participants described experiencing were mainly perpetrated by other children and adolescents, in particular, classmates (some of whom were their friends), and other children at school. Some participants also described experiencing negative treatments from people in their communities, and some family members, although, in general, family members as well as friends were reported as the ones who would support them when others treated them negatively because of their conditions. Participants across health conditions also described a few instances of negative and discouraging interactions with teachers and healthcare professionals because of their conditions that could be considered stigmatizing.

Here, participants across conditions described negative interactions with their teachers that could be considered stigmatizing:

Like, one of my PE [physical education] teachers...…, she didn’t believe. Like, one day I was kinda [kind of] having an attack. So, my attacks come in the form of, it makes you, it feels like a cold, but it’s not.....and it makes your chest feel tight. So, because I was coughing, I told her that, you know, my chest was feeling tight, and the medication wasn’t really working. I told her I don’t feel like I can run, so if she can give me a pass to go to the nurse …. And she refused. But a next teacher who knows the case, because they actually document it that I do have asthma, on my file.... she came over, and she was like, “No, this kid is really ill,” and all of that. And she was the one who actually got me to go to the nurse ….. to get treatment.

(*Female, 15 years old, Pulmonary condition – Asthma*)

Mi [I] can... rememba [remember] dis [this] one day, the teacher come class – that was Spanish class…. Suh [So],….. she start teach. Now, mi [my] blood sugar guh [went] low. Mi [I] tell har [her] seh [that] mi [my] blood sugar low, which she never know seh [that] mi [I] have diabetes, suh [so], she nuh [don’t] really understand. ….. So, mi [me] a tell har [her] mi [me] have sugar, shi [she] seh [say] mi [me] fi [must] gwaan [go on] a [to] mi [my] seat. Suh [So], mi [me] deh deh [right there] a shake, shake. Mi [Me] tell har [her] and shi [she] still... nuh [don’t] pay mi [me] nuh [no] mind. Suh [So] mi [I] …. run out a [of] di [the] class... and di [the] same youth weh [that] come with mi [me], him …… ask mi [me] wha [what] duh [happen to] mi [me]. Mi [Me] tell him mi [my] blood sugar low. Him tek [take] out one juice outta [out of] him bag, him did just buy... [When] mi [I] guh [go] back a [to] class now, shi [she] a tell mi [me] sorry. Mi [Me] tell har [her] seh that’s alright.

(*Male, 16 years old, Endocrine condition – Diabetes*)

Here are participants’ descriptions from across conditions of their interactions with healthcare professionals that could be considered stigmatizing:

Yeah, [I believe that I am fat]...but when I see other people, I say, “But that is obesity.” But… the doctors at this clinic...they are the ones that actually make me believe that I am fat... Especially when they use the term obesity…. [They say I am] obese.

(*Female, 15 years old, Endocrine condition – Weight issues & Hormone imbalances)*

well, sometime mi [I] don’t like when some doctor know…Because from mi [I] mek [make] dem [them] know, dem [them] scorn mi [me]. That’s why sometime mi [I] don’t like tell dem [them] mi [my] business.... di [the] one dem [them] weh [that] wear di [the] blue run mi [me], call mi [me] battyman and dem [those] tings [things].

(*Male, 17 years old, HIV*)

### Rejected/Resisted stigmatization

Besides accepting stigma through limiting disclosure about their health conditions, avoiding social interaction and experiencing various negative feelings and beliefs about self, participants across conditions also described instances where they tried to resist or reject the stigma they perceived or experienced. They rejected the stigma in various ways, including adapting their thinking about their experience (reframing their experience), seeking support from friends or family, educating others or self about the condition, and proving people wrong. Table 4 below provides illustrative quotes for each way the adolescents reject stigma.

**Table 4:** Sample illustrative quotations for each way the adolescents rejected stigma.

Ways of rejecting stigma	Illustrative quotations across health conditions
**Reframing experience**	[Students in my class] dem [them] know seh [that] mi [I] come [to the clinic].... Dem [They] respond to me normal like how anybody else woulda [would] respond to me.... Sometime …. dem [they] woulda [would] trouble mi [me] and seh [say], “Hey [Hello] mad bwoy [boy],” or suh [so]… Is just… suh [so] wi [we] live.... Like, …. if yuh [you are] blind or suh [so], dem [they] seh [say], “You blind bwoy [boy] come yah [here],” becah [because] mi [I] do some things, sometime …… out of proportion. (*Male, 14 years old, Mental illness*) my friends …. have dem [them] time when dem [them] …… call mi [me] like, Sugar, Sweet Sugar, Sweetie … Because the other word for diabetes, old people call it, suga [sugar]… I just laugh [when they call me those names] because by saying sweet, sweetie and suga [sugar], people nah [not] guh [going to] know. Because Jamaican people have it to seh [say] yuh [you] call yuh [your] fiancé or your spouse sweet or sweetie or sugar. Suh [So], dem [them] nah [not] guh [going to] click and think that extreme like, fi [to] know seh [that] mi [I] have diabetes. That’s why dem [them] a call mi [me] suh [so]. (*Female 1, 15 years old, Endocrine condition – Diabetes*)
**Support from family or friends**	some comments thrown out there, probably not meaning to hurt … for some reason, hurt me. Like, the fact when some people are talking about how much weight people have on…and constant judging. And they talk about people that probably most of the times fall into the range of my size. And I am thinking, “What if I wasn’t friends with them? Probably those words would be thrown on me.” I mean, there is a group at school that doesn’t like me, but my friends are there to keep my hopes up. Just there to encourage me, like, “Hey, you look better than them.....Let them talk what they want to talk. Yes, you might have on a bit of weight that you don’t want, but at least you look good with it.” So, that is their strategy. (*Female, 14 years old, Endocrine condition – Weight issue & Early cycle)* Alright, [my aunt] treats me different from my sisters. So, there is some jealousy right there.... shi [she] treat all of us the same, you know. But.... tru [because] shi [she] see that my sisters, they are against mi [me]...and everything… she is more harsh with them... Like, “Why can’t you live in peace with your sister?” (Female, 15 years old, Neurological condition – Epilepsy)
**Educating others or themselves about condition**	I couldn’t do anything at the time [when they said I was faking] because I was having really bad chest pains. So, I didn’t, yeah, I couldn’t do anything at the time. But, like when I got back to them, I was like, “Are you serious!” I asked them if they were crazy. They should go and look it up on the internet or something if they have any sense. … because I [was] like, “How can you say that to someone when you have never been in their position? You don’t know what it is like to kinda [kind of] just stop breathing, you can’t breathe and all that. You don’t know how stressful it is or what you can and cannot do with it.” (*Female, 15 years old, Pulmonary condition – Asthma*) [My family], they mek [make] it look like I cause it on myself. Most of them cuss [curse] mi [me] and tell mi [me] a lot of things, like, I eat too much sweets.… Like, one point, …. [I believed what they said] …. until I start researching for myself about diabetes.... And I found out that it’s not my fault. (*Female, 18 years old, Endocrine condition – Diabetes*)
**Proving family members and others wrong**	[Yeah, it is better if less people know]. Because sometimes, if dem [them] know, dem [they] a [are] guh [going to] treat me like mi [me] sick and mi [I] can’t do anything.… Once. … like my aunty came from England…. she asked me to move something and….. somebody said, “No, him can’t move it, him sick.” …. [But]…, I actually moved it. It wasn’t any pressure. (*Male, 16 years old, Sickle Cell Disease*) [When they would say that I am half-crazy], I proved to them that I wasn’t … How I prove it now? Hmm, like having one and one discussion, just like general … that make me more of a, you know, somebody easy to talk to about topics [from a] reasoning point level. Yes, that’s it.... how I try to say, to prove to them I wasn’t mad – based on my level of reasoning…They wouldn’t actually pick up to that point that I actually am that. (*Female, 18 years old, Mental illness*)

## Discussion

This study sought to examine personal experiences with individual-level stigma – perceived, internalized and enacted stigma – among adolescents with diverse chronic illnesses, with the intention to elaborate the elements that make up each form of stigma among adolescents that are common across conditions. Through using the accounts of Jamaican adolescents, the elements of the different forms of individual-level stigma as commonly experienced by adolescents in the context of their daily lives were identified. A number of the elements identified for the different forms of stigma are similar to those that have been described in the adult literature; however, there are those that seem unique to adolescents and their developmental level. Conducting the qualitative research better allowed for these unique elements to be identified.

### Adolescents’ experience of perceived stigma

The adolescents’ experiences of perceived stigma mainly involved an expectation of various negative treatments due to their illness that included a fear that they would be rejected by their peers in a series of specific ways, a fear that their condition could be used against them, and a fear that discussing their condition may result in them being rejected by peers and important adults. Their awareness of stereotypes regarding their specific illnesses was limited and based on personal experience or witnessing how others were treated. Additionally, as the adolescents got older, they appeared to identify specific features of a stereotype regarding their condition.

The adolescents’ experience of perceived stigma is unlike what is theorized for adults in the modified labelling theory [42]. In the theory, it is suggested that there is equal influence on perceived stigma of anticipation of stigmatization due to one’s illness and having an awareness of the stereotypes regarding the illness. However, for the adolescents in this study, experience of perceived stigma was more focused on their anticipation of negative reactions or treatments if others knew about their illnesses, and less so on having an awareness of societal stereotypes and attitudes regarding their illnesses. Child development may explain the lesser influence of awareness of illness-specific stereotypes on the adolescents’ experience of perceived stigma. As younger adolescents have not fully developed their skills in abstract reasoning, they may not be able to describe or understand a stereotype about their specific condition. However, as older adolescents are more developed with expanded cognitive skills, they would be more aware of a stereotype about their condition, but this awareness would not be as elaborate as it is for adults [48,84].

### Adolescents’ experience of internalized stigma

Regarding self or internalized stigma, the adolescents internalized their expectation of negative treatment, which resulted in a strong need to limit disclosure about their conditions and to avoid social interaction with others. Additionally, those adolescents who were aware of societal stereotypes regarding people with their conditions tended to internalize elements of the stereotypes, and these manifested as various negative feelings and concerns about themselves.

Several of the aspects of internalized stigma that were identified as part of the adolescents’ experience (e.g., kept things about the condition a secret, avoided social interaction, felt embarrassed, felt different, felt left out) are similar to those that have been reported in the adult literature [42,85–88]. However, the adolescents’ experience of internalized stigma also included aspects that seemed unique to them and their developmental level, for example, the elements of wished normal or healthy, or felt they were weak because of their condition. Although adults could also experience these elements of internalized stigma due to their illnesses, they are more salient for adolescents due to the likely negative consequences for their social and emotional development, and hence, their psychosocial outcomes. As noted in literature, peer relationships and acceptance, and a growing sense of self and personal identity are key areas for adolescents’ social development [22,47] Hence, having to cope with a long-term illness that is stigmatized, adolescents would likely be very sensitive to any way they are different from their friends and other peers (e.g., not feeling normal, can’t do things like others do). This may lead to them engaging in various avoidance activities that may affect their peer relationships and identity formation, which in turn may affect their self-esteem and mental health [89].

### Adolescents’ experience of enacted stigma

Enacted stigma (negative treatments) was experienced in a variety of ways by the adolescents, both overtly and subtly, and mainly in their interpersonal relationships. The various negative treatments were perpetrated mainly by other youth at their schools, although friends, family members, teachers, and healthcare professionals also treated them negatively.

Like aspects of internalized stigma, a number of the negative treatments the adolescents experienced are similar to those reported in the adult literature, for example, being called names, pitied, treated as different, and gossiped about [86,90,91]. However, there are those negative treatments that seem unique to adolescents, such as being treated like a baby, or treated softly; being accused of faking; or told things about their condition to make them worry, fret or feel afraid. Again, adults may have some or all these experiences or equivalents, but they are more salient for adolescents, given their critical stage of identity and social development [22,92].

The adolescents’ interpretation of some of the treatments they received from others as negative differed among them. Some treatments were seen as negative by some adolescents, while others viewed them positively (e.g., being babied/treated softly, being pitied). Even well-intentioned actions of others (e.g., checking on adolescents or monitoring their behaviours due to their condition) were seen as negative by some adolescents.

One explanation for why the adolescents differed in their interpretations of some of the treatments they received from others could be their awareness of the negative features of the stereotype that has been associated with them due to their specific illness. Literature on social identity threat suggests that an awareness of the negative aspects of a stereotype is needed to interpret an ambiguous action as an instance of enacted stigma [41]. As not all of the adolescents may have been fully aware of the negative features of the stereotype that had been associated with them due to their condition, this may explain why some of them interpreted an action such as being babied negatively, while others interpreted it positively [41].

### The influence of cognitive and social development

The results of this study also showed that the adolescents’ experience of the different forms of stigma varied over the course of their health conditions due to their cognitive and social development. In terms of cognitive development, the literature indicates that as children move into adolescence, they move from thinking in concrete terms to being able to think more abstractly and take another person’s perspective [17,48]. These changes in cognitive capabilities will affect how adolescents interpret the experiences they have had as well as their ability to express these experiences or provide their opinions about the experiences. The expansion of the cognitive ability of adolescents allows them to reflect upon their experiences and express their internal states in both emotional and non-emotional ways. They gain the ability to interpret their experiences rather than simply describe them. The influence of cognitive development was seen in the adolescents’ experiences with stigma changing over the course of their illnesses due to cognitive maturity or growth.

The influence of social development was seen in terms of the adolescents’ transitioning from one school level to another (e.g., from primary school to secondary school); the rise in importance of peer groups to their perception of self; and the adolescents taking steps, such as secrecy to reduce negative experiences. During adolescence, the importance of fitting in and belonging to a group of similar peers emerges. As indicated in the literature, as children move into adolescence, they shift their focus from the importance of interactions with their parents and family to interactions with peers [22,47,89]. Consequently, the issues of fitting into a social group and gaining peer acceptance become very salient. Experiencing an illness, by its very nature, sets adolescents apart from their peers. Consequently, issues of normalcy, secrecy, limiting social interaction, and expectation of negative treatment also become salient as adolescents’ management of their illness will likely be at odds with their normal developmental strivings [93,94]. Therefore, the emergence of these aspects within the sample of adolescents could be a product of their social development. It is through the developmental task of integrating oneself with peers, along with the increased cognitive capabilities of being an adolescent, that the desire to be normal like others, issues of secrecy, isolation, and fear of negative treatments become strong enough to be a part of their experience of stigma.

### The influence of culture and society on stigma experience

The experiences of stigma among the adolescents in this study were significantly shaped by the interplay between their chronic illnesses and specific socio-cultural norms within the Jamaican society. Two major cultural aspects—gender roles and parenting practices—influenced how the adolescents experienced stigma.

#### Gender roles and stigma experience.

The adolescents reported gender differences in how they perceived and experienced their stigma due to their health condition. Male adolescents were reluctant to discuss their condition or ask doctors questions. This behavior can be interpreted through the lens of Jamaican socialization, which promotes a “macho” identity [53]. This cultural ideal, emphasizing strength and toughness, encourages males to suppress problems, thus avoiding a direct contradiction of the expected image of physical fortitude. Conversely, female adolescents, especially older ones, feared rejection from male peers if they disclosed their condition and reported feeling limited or weak. The illness challenged the salience of body image during adolescence, leading to concerns about being less attractive and capable than peers [95]. The illness thus converts a medical reality into a source of cultural stigma by challenging the expected capabilities for both genders.

#### Parental practices and stigma experience.

Adolescents reported that their parents often restricted their activities, constantly monitored their behaviour, and checked on them due to their health conditions. They perceived these actions as overprotective and intrusive, which irritated them. This pattern aligns with the cultural tendency of Jamaican parents to remain highly involved in their adolescents’ lives, often well into adulthood [57]. This extensive parental monitoring, while intended to protect, inadvertently contributed to the adolescents’ experience of enacted stigma. By marking the adolescents as needing special protection and restriction, the practice reinforces their sense of being different or incapable compared to their peers, solidifying the social distinction created by the chronic illness. This cultural context is essential for readers outside Jamaica to grasp the complexity of the participants’ experiences.

### How the modified labelling theory and its expansions fit with the stigma experience of the adolescents

The results of the research provide support for several of the premises of the modified labelling theory and its expansions. The results showed that the three forms of individual-level stigma, that is, perceived, internalized and enacted stigma, which are highlighted in the theory, are experienced in a general way across illnesses in the adolescents who participated in the study. The adolescents reported experiences that were common across multiple conditions and could be categorized into the three forms of stigma. However, only partial support was found for their experience of perceived stigma.

The results showed that the adolescents’ experience of perceived stigma was centered on their anticipation of various negative treatments due to their illnesses, rather than also having an awareness of the negative societal stereotypes related to their conditions, as suggested for adults by the Modified Labelling Theory [42]. In fact, the adolescents’ awareness of stereotypes regarding their illnesses was limited. This lack of awareness of the illness-specific stereotypes among the adolescents suggests that perceived stigma likely occurs through a different mechanism for them than for adults. The theory suggests that having an awareness of negative stereotypes about one’s illness is a central feature of the experience of perceived stigma as an adult [42]. It also suggests that awareness of the negative stereotypes grows prior to adulthood; however, there is little suggestion of the mechanism through which this occurs. The mechanism through which this occurs could be cognitive and social development. Based on the literature, during adolescence, young people begin to be able to think and reason abstractly, which will allow them to identify commonalities between basic facts and experiences [17,18,48]. As well, during this period, young people begin to compare themselves to others and to understand social norms. It is both the growth that is happening in abstract reasoning and the social comparison processes during adolescents’ development that will enable the development of knowledge regarding health-related stereotypes, which is the basis of adults’ awareness of stereotypes. As such, it may be suggested that the modified labeling theory should be further expanded to include how cognitive and social development serve to bring about the knowledge of a stereotype.

### Implications of the findings

#### Interventions for schools and families.

The findings indicated that enacted stigma perpetrated by peers is a primary source of negative influence on adolescents’ experiences of perceived and internalized stigma. Therefore, interventions are critically necessary in schools to address this issue at its most frequent point of occurrence. These programs must be implemented to sensitize all students about their stigmatizing behaviors toward peers with health conditions and educate them on the harmful psychosocial effects of these actions. Furthermore, separate, tailored interventions should be developed for parents and teachers, who were also identified as sources of enacted stigma, to promote supportive and non-judgmental environments.

#### Healthcare system sensitivity.

Since healthcare professionals were also implicated as sources of stigma, health clinics serving children and adolescents should prioritize sensitivity training for staff. This training should specifically address how the language, non-verbal communication, and reactions of clinic staff influence the young client’s experience of stigma, perceived social support, and willingness to disclose information about their condition.

#### Policy and program development.

The research also provides a framework for developing policies aimed at comprehensive stigma reduction. Policy makers should recognize that the profound impact of a stigmatizing act is not solely determined by its objective severity or reprehensible nature, but often by the adolescent’s subjective interpretation of that act. Because a seemingly innocuous or culturally accepted comment can cause profound harm, policies should instruct program developers to adopt a broad, context-aware approach, targeting all forms of communication and behavior that may be subjectively experienced as invalidating or harmful by youth with chronic conditions.

### Strengths of the study

The results of this qualitative study make a significant contribution to the literature by adding information on how adolescents with diverse health conditions commonly experience the forms of individual-level stigma. As highlighted in the literature review, the experience of stigma is a subjective one, and measurement of it is better informed by the lived experiences of the targets. This is the first known study to examine the common ways stigma is experienced across health conditions in youth.

### Limitations of the research and recommendations for future work

Although the study has some strengths, it also has a few limitations that need to be highlighted. First, due to the use of convenience sampling to conduct the study, the full range of commonalities across conditions may not have been identified. Had purposive sampling been used, then a larger number of commonalities across the conditions could have been identified.

A second limitation is that the health conditions of the adolescents who took part in the study were largely hidden from others, meaning that their symptom manifestation was often not visible to the public. The commonalities identified may not be generalizable to adolescents with more visible and more serious manifestations of illnesses. Future research should include adolescents with more visible manifestations of their illnesses. Additionally, as a convenience sample of Jamaican adolescents was used in the study, the findings may not be generalizable to adolescents across Jamaica, the Caribbean, or internationally. Future research should target more representative samples of adolescents from Jamaica as well as adolescents from other cultures. Even further, as the findings presented in this paper were based on data collected nearly ten years ago, the study should be replicated to determine how consistent and generalizable the adolescents’ experiences are.

A third limitation is that youth younger than 12 years of age were not included in the research. As such, the findings may be limited only to adolescents and may not represent the stigma experienced by children with illnesses. For a number of reasons, the experience of stigma for children may be different from that for adolescents. Children may not have had the cognitive abilities to make sense of their experiences. As well, the social experiences of children are different from adolescents, meaning that there may be aspects of stigma experienced by children that would not be captured in a sample of adolescents. Work should be undertaken to expand the range of ages and experiences to which the measures may be applied.

## Conclusions

The study found that the adolescents’ experience of the different forms of individual-level stigma bore some similarities with adults. Also, there were differences identified in their experience of stigma from adults. Many different things could explain the differences between adolescent and adult experiences of stigma across conditions. One possible explanation for these differences may be the influence of social and cognitive development. As such, it may be suggested that the influence of social and cognitive development be incorporated in how perceived, internalized and enacted stigma develop at the individual-level in adolescents with an illness. Another implication of the study is that the findings should be considered in quantitative assessments of individual-level stigma in adolescents with an illness.

## Supporting information

S1 File In-depth interview guide. (PDF)

S2 Table Jamaican dialect and English translations. (PDF)
