# Supplementary material for: A qualitative exploration of the experience of individual-level stigma among adolescents with a chronic illness
Source: PLoS One. 2025 Dec 18;20(12):e0317633. doi: 10.1371/journal.pone.0317633 (PMC12714286; doi:10.1371/journal.pone.0317633)
Supplement: S1 File — (PDF) [file pone.0317633.s001.pdf]

In-depth interview guide

Hello (*insert participant's name*).

Thank you for agreeing to allow me to talk to you. I would like to ask you some questions on your thoughts and feelings about having [*insert condition*] and things that might have happened to you because of it. There are no wrong or right answers. Remember, you do not have to answer any question that makes you uncomfortable. If there is anything you do not understand, please tell me and I will explain it to you. If you want me to repeat anything, tell me and I will repeat it for you.

I will be tape recording what we talk about today. This is so that I can get all of what you say and be able to keep talking to you at the same time. After we are finished, I will listen to the tape and write out what we talked about. However, you don't have to worry that anyone will find out what you said as your information will be kept private, and your name will not appear in the text.

Halfway through the interview I will give you a 15-minute break.

To begin I would like to ask some questions about you and your family.

Demographic information

1. How old are you?
2. When were you born?
3. Who is the main person who looks after you? Do you live with this person? (If no, probe: Who do you live with?)
4. Who else lives at your home? (Probe: brothers & sisters, relatives, other people, & numbers)
5. What school do you attend? What grade are you in? (If not in school, obtain last school & grade)

Discovery of Health condition

1. How did you find out that you have (*insert condition*)? (Probe: Who told you*?* When? Age found out? How did you feel when you found out?)
2. Do you have any other condition(s) along with the (*insert condition*)? (If yes, probe: What? Which condition affects youth more?]
3. What do you think has caused you to get (*insert condition*)? (Probe: Who*?* Where? etc. How did you feel when you found out?)
4. What are the symptoms / problems that you get with having (*insert condition*)?

Treatment & Adherence

1. Do you always come to this doctor/clinic for your (*insert condition*)? (If no, probe: Where else do you go? Why?)
2. How do you feel about coming to the clinic? (Probe: What do you like / dislike about clinic? How do you get along with the doctors / nurses who look after you? How do you feel about the way they treat you? Anyone done or said anything you did not like?)
3. Has the doctor given you medication to take for your (*insert condition*)? (If yes, probe: What? How often are you to take it? When do you take it? Where? How do you feel knowing that you have to take medication? etc.)
4. What else has the doctor / nurse told you to do to take care of your (*insert condition*)? (Probe: Have you been told not to eat certain foods? To exercise? etc.)
5. Do you have/ Have you ever had any problems with taking the medication or doing the other things the doctor/nurse tells/has told you to do? IIf yes, probe: What? Do you do it? etc.)
6. Do you ever talk to the doctor/nurse about any of the problems you have? (If yes, probe: What do you talk to them about? What does he/she say? What does he/she tells you to do?)
7. Do you take/ Have you taken other kinds of things for your (*insert condition*)? (If yes, probe: What? Why?)
8. Have you ever been admitted to the hospital for your (*insert condition*)? (If yes, probe: When? What were the reasons? Etc.

Disclosure about condition

1. Who are the people that know that you have (*insert condition*)? Do they know that you come to the clinic, take medication, etc? (Probe: family members, friends, people in community and at school know? How did they find out? How did they react? Why do you think they reacted that way? How did it make you feel?)

Others Views and reactions towards people with condition

2. How do you think other people treat someone who has (*insert condition*)? (Probe: Do you think they treat them differently from those who do not have the condition? If yes, probe: In what way/s?)
3. Have you heard other people say things about someone with (*insert condition*)? (It can be good or bad). [If yes, probe: What? Who? When? Where? Why? Do you hear them call them names? What? Has this affected you in any way? How?)
4. Have you seen things on the television / heard things on the radio / read anything in a book or anywhere about people with (*insert condition*)? (If yes, probe: What? When? Where? etc, Has this affected you in any way? How?)
5. Have you had any negative or bad reactions from or with anyone because of your (*insert condition*), medication, etc? (If yes, probe: What? Who? etc. Types of reactions: reactions: teased, made fun of, avoided, mean things said to you, blamed, someone acted uncomfortable around you, called names, insulted, treated unfair, etc.)
6. Do you think that anyone treats you differently / acts differently towards you - it can be good or bad - because they know you have (*insert condition*)? ([If yes, probe: Who? In what way/s? How does it make you feel? What do you do when this happens? If no, probe: Why do you think no one has? Parents/guardian treats you differently from siblings? Teachers treat you differently from classmates? Friends treat you as if you are different from them?)

Feelings about condition

7. Do you know of anyone your age / any other young person who has (*insert condition*)? (If yes, probe: Who? Do you talk to them about (*insert condition*)? If no, probe: Does it bother you that you do not know anyone? How?)
8. How do you feel about having (*insert condition*)? (Probe: Does having (*insert condition*) or having to manage the symptoms ever upset you or get you down? (If yes, probe: Tell me about it.)
9. Does anything worry / bother you about having (*insert condition*)? (If yes, probe: What? Why? How do you handle it? Do you share it with anyone?)
10. Does having (*insert condition*) and / or having to manage it (e.g., deal with symptoms, come to the clinic, etc) ever affected how you feel or think about yourself? (If yes, probe: In what way/s?)
11. Does it matter to you / bother you what people might think if they find out about your (*insert condition*)? (Probe: Why / Why not?)
12. Do you think that having (*insert condition*) make you different from other children/young people? (If yes, probe: In what way/s? Why?)
13. Do you feel that you are inferior to other children / young people because you have (insert condition)? (If yes, probe: In what way/s? Why/Why not?)
14. Do you ever feel embarrassed about having (*insert condition*)? (If yes, probe: What? etc.)
15. Do you ever blame yourself for anything regarding your (*insert condition*)? (If yes, probe: What? Why? Do you share these feeling with anyone?)
16. Do you think that having (*insert condition*) has spoiled your life? (If yes, probe: In what way/s? How would your life be different if you did not have condition?)
17. Do you act differently around anyone because you don't want them to find out about your (*insert condition)*?
18. Who would you ask for help if you had a problem in coping with your (*insert condition)*? (Probe: people at home and school - family members, friends, classmates, etc.)
19. Do you ever try to hide or keep as a secret from others certain things about your (*insert condition)*? (If yes, probe: Who? What? – taking meds. etc.
20. Do you keep to yourself/stay by yourself/avoid certain situation because of how you think other people may react if they find out about your (*insert condition)*? (If yes, probe: Tell me about it.)

Effects of condition on your life

21. How has your (*insert condition*) affected your life? Probe as necessary
22. Do you have any problems with family members/friends etc. because of your condition? (If yes, probe: what?
23. Do you have to do anything differently because of your (*insert condition*)? / Does having (*insert condition*) interfere with you doing anything / any activity at home, school, etc.? (If yes, probe: What? How does it make you feel? What do you do instead?)
24. Is/Are there any activity/ies that your family or friends leave you out of because of your condition?)
25. Is/Are there any activity/ies that your parents / teachers (if know) try and stop you from doing because of your condition? [If yes, probe: What? Does it bother you that you are not able to do these things? What do you do instead?)

Other questions

26. Does your school have any service/s to help children with (*insert condition*)? (If yes, probe: What? How did you hear about it? Have you ever used it? Did you like it? [If no, probe:] What do you think your school can do to help children/young people with (*insert condition)*?
27. What is the most difficult or hardest thing for you about having (*insert condition*)? Can you think of anything good that has come out of you having (*insert condition*)? (If yes, probe: What?)
28. What do you think the clinic can do to help you deal with your (*insert condition*) better?
