# Supplementary material for: A qualitative exploration of the experience of individual-level stigma among adolescents with a chronic illness
Source: PLoS One. 2025 Dec 18;20(12):e0317633. doi: 10.1371/journal.pone.0317633 (PMC12714286; doi:10.1371/journal.pone.0317633)
Supplement: S2 Table — (PDF) [file pone.0317633.s002.pdf]

Table. Jamaican dialect and English translations

Words	Translations
a	Is/are/to/of
Affi	Have to
‘ave	have
Bout	about
Bredda	brother
Bruk up	break
bwoy	boy
Cah	because
coulda	Could / could have / could of
cyaan	cannot
cuss	curse
Dah	this
Dan	than
Deh/dey	They
Deh deh	Right there
dem	Them/they/those
Dere	there
Di	the
Dis	this
Duh	Happen to
Fi	To/for/must
grandmadda	grandmother
Gwaan	Go on
Guh	go/Going to
Har	her
inna	into
Jook	stick
ketching	catching
Kinda	Kind of
Madda	mother
Mawga down	Meager/skinny
memba	remember
Mi	Me /I/my/ I am
Myself	myself
nah	not
nuff	Plenty / a lot of
nuh	Don’t/doesn’t/no/not
Nuh mind	Any attention
One bag a summen	One bag of Something/things
Outta	Out of
Rememba	remember
seh	Say/said/that / saying

**Words**	**Translations**
shi	she
Si	see
suh	so
suga	sugar
Summen/someting	something
tek	take
Tenda	tender
Tru	because
Wata	water
Wha	what
weh	that
Wi	We/us
Woulda	Would of / would have/would
Yah	here
yuh	You/your/you are
